# Acoustic and Language Based Deep Learning Approaches for Alzheimer's Dementia Detection From Spontaneous Speech

**DOI:** 10.3389/fnagi.2021.623607

**Published:** 2021-02-05

**Authors:** Pranav Mahajan, Veeky Baths

**Affiliations:** ^1^Cognitive Neuroscience Lab, Department of Electrical and Electronics Engineering, BITS Pilani University K. K. Birla Goa Campus, Pilani, India; ^2^Cognitive Neuroscience Lab, Department of Biological Sciences, BITS Pilani University K. K. Birla Goa Campus, Pilani, India

**Keywords:** affective computing, cognitive decline detection, natural language processing, deep learning, computational paralinguistics

## Abstract

Current methods for early diagnosis of Alzheimer's Dementia include structured questionnaires, structured interviews, and various cognitive tests. Language difficulties are a major problem in dementia as linguistic skills break down. Current methods do not provide robust tools to capture the true nature of language deficits in spontaneous speech. Early detection of Alzheimer's Dementia (AD) from spontaneous speech overcomes the limitations of earlier approaches as it is less time consuming, can be done at home, and is relatively inexpensive. In this work, we re-implement the existing NLP methods, which used CNN-LSTM architectures and targeted features from conversational transcripts. Our work sheds light on why the accuracy of these models drops to 72.92% on the ADReSS dataset, whereas, they gave state of the art results on the DementiaBank dataset. Further, we build upon these language input-based recurrent neural networks by devising an end-to-end deep learning-based solution that performs a binary classification of Alzheimer's Dementia from the spontaneous speech of the patients. We utilize the ADReSS dataset for all our implementations and explore the deep learning-based methods of combining acoustic features into a common vector using recurrent units. Our approach of combining acoustic features using the Speech-GRU improves the accuracy by 2% in comparison to acoustic baselines. When further enriched by targeted features, the Speech-GRU performs better than acoustic baselines by 6.25%. We propose a bi-modal approach for AD classification and discuss the merits and opportunities of our approach.

## 1. Introduction

Alzheimer's disease and related dementia disorders constitute a significant cause of disability and dependency among older adults worldwide and are among the costliest diseases in society. By 2030, it is estimated that the global cost of dementia could grow to US$ 2 trillion, which could overwhelm health and social care systems (Wimo et al., 2017). Alzheimer's Dementia (AD) is an irreversible brain disease that results in a gradual decrease in an individual's cognitive functioning. The main risk factor for AD is age, and therefore its highest incidence is amongst the elderly. However, if detected early, we can slow down or halt the degeneration with appropriate medication. Current methods of diagnosis usually involve lengthy medical evaluations, including lengthy questionnaires. There is an urgency for cost-efficient and scalable methods that can identify AD from an early stage. Thus, researchers worldwide are trying to find non-invasive early detection methods and treatments for these disorders.

Early symptoms of dementia are characterized by difficulty in word-finding, impaired reasoning, changes in language and speech, etc. This makes current research methodologies in speech and language processing suitable to be applied for early detection of cognitive impairment and AD. AD detection from spontaneous speech has been approached using speech input-based methods, language-based (text input-based) methods, and multi-modal approaches. Deep learning is a part of a broader family of machine learning methods based on artificial neural networks with representation learning. In prior work using language-based methods, we observe that deep learning based approaches (Orimaye et al., 2016; Karlekar et al., 2018; Di Palo and Parde, 2019; Kong et al., 2019) outperform pre-deep learning approaches (Orimaye et al., 2014; Fraser et al., 2016) on the DementiaBank dataset (Becker et al., 1994). Motivated by the shortcomings of manual feature-engineering for such a diverse and complex task, Karlekar et al. (2018) propose deep learning models—Convolutional neural network (CNN), Long short-term memory network (LSTM), and CNN-LSTM, to detect AD using just the conversational transcripts with minimal feature engineering using just word embeddings and parts-of-speech (POS) tags. Word embedding is any set of language modeling where words from a vocabulary are mapped to a vector of real numbers. POS-tagging is assigning a parts-of-speech tag to every word in the corpus, depending on it's context and definition. It is a method of enriching the feature processing stream. CNN layers are locally connected layers and pick up features in shorter time windows, where as LSTM layer is a type of recurrent neural network (RNN) layer which learns features and remembers features over longer timesteps. Recurrent layer or recurrent unit is any layer whose output not only depends on the input at the current timestep but also it's hidden state in the previous timestep. Thus, a CNN-LSTM architecture uses convolutional layers early on for feature extraction and then LSTM layers to learn patterns in a sequence. Di Palo and Parde (2019) further enrich the deep neural network models by Karlekar et al. (2018) by using targetted psycholinguistic, sentiment, and demographic features and also use class weight correction to handle class imbalance in the DementiaBank dataset (Becker et al., 1994). We build upon the work by Karlekar et al. (2018) and Di Palo and Parde (2019) and extend to multi-modal inputs and address the challenges that come with effectively combining features from multiple modalities for AD detection.

Amongst speech input-based methods, prior work has been more focused on using handcrafted acoustic features (Beltrami et al., 2016; Ambrosini et al., 2019) such as pitch, unvoiced duration, shimmer, pause duration, speech rate, or using feature banks. Haider et al. (2019) and Luz et al. (2020) use feature banks such as such as emobase, eGeMAPS (Eyben et al., 2015), ComParE (Eyben et al., 2013), and MRCG functionals (Chen et al., 2014) for feature extraction from speech segments. These features are not necessarily designed specifically for AD speech but capture various paralinguistic features relevant to AD speech. Effectively combining these features from various speech segments is an ongoing research problem that our work addresses. Previously, Haider et al. (2019) address it by proposing a new Active Data Representation method (ADR) to combine the features from a variable number of recordings into a fixed dimensional feature vector. They get the best results using the eGeMAPS feature set and even better results using a hard fusion of the feature sets. However, these methods fail to capture the temporal dynamics across the segments to the full extent. In this work, by using a recurrent unit, we combine the speech segment features in a fixed dimension vector while learning the features across the time span of the participant's conversation session. Chien et al. (2019) implement a bidirectional RNN on speech features extracted using a feature bank and propose an end-to-end method for automatic assessment of cognitive decline, but are restricted to speech input and do not extend to multi-modal inputs. Amongst multi-modal approaches using spontaneous speech, Zargarbashi and Babaali (2019) propose a model that extracts a perplexity score from the transcripts using an N-gram model extract I-vectors and X-vectors from the speech input. The concatenation of these feature vectors is then passed on to an SVM for AD classification. X-vectors and I-vectors are speech embeddings used in speaker recognition tasks, especially with speech segments of variable lengths. They use these embeddings even though AD diagnosis and speaker recognition are different tasks, as the voice biometrics and Alzheimer's signs are similar to an extent as both need to extract some specific patterns from captured signal contaminated with variations from various irrelevant sources. This prior work mentioned is relevant to our work because our work focuses on some of the open research problems, such as—How to capture complex patterns and temporal relations in speech and language modalities? And more importantly are there temporal patterns in the acoustic features extracted using the feature sets mentioned above, which can prove to be useful early detection of AD.

The majority of the previous results have been benchmarked on subsets from the Cookie theft task from the DementiaBank dataset (Becker et al., 1994) except the work by Chien et al. (2019) where they use NTUH Dataset which is a combination of multiple datasets such as Mandarin_Lu dataset (MacWhinney et al., 2011), NTU dataset (Chien et al., 2019), and 20 more participants from independently collected data. Dementia Bank dataset includes multiple transcripts from the same participant and has a significant imbalance in the age and gender distribution of the participants. ADReSS dataset (Luz et al., 2020) tries to mitigate these issues, and thus we use the ADReSS dataset in our work.

In this work, we address this by proposing a network that can train on speech segments using recurrent units and can be integrated with existing language-based deep learning models, which can also be enriched with targeted features.

Our contributions are as follows:

1. We re-implement the prior work by Karlekar et al. (2018) and Di Palo and Parde (2019) and benchmark the results on the new shared standardized ADReSS dataset.
2. We explore the deep learning-based methods of combining acoustic features into a common vector using recurrent units and propose a bi-modal approach for both the tasks.
3. We discuss the possibilities of further enriching the acoustic processing stream using features specific to AD speech and propose a bi-modal model based on concatenation of latent outputs of acoustic and language based models.

## 2. Materials and Methods

### 2.1. ADReSS Dataset

Most earlier methods use a subset of the DementiaBank (Becker et al., 1994). Cookie theft task provides the largest source of unstructured speech and text data and thus has been used in Karlekar et al. (2018), Di Palo and Parde (2019), and Kong et al. (2019). The subset used in Di Palo and Parde (2019) includes multiple transcripts from the same participants, thus comprises a total of 243 transcripts from 104 non-AD participants and 1,049 transcripts from 208 AD participants. It also has imbalances in age and gender distribution. ADReSS Challenge dataset (Luz et al., 2020) tries to mitigate these issues. ADReSS Challenge dataset includes one full-wave audio (one session) per subject with accompanying conversational transcript. It also has a balanced distribution in terms of classes, age, and gender. As a result, we notice more than ten times reduction in dataset size in terms of the number of transcripts or full-wave session audios when compared to the dataset used in Karlekar et al. (2018), Di Palo and Parde (2019), and Kong et al. (2019). This is important to us since deep learning methods proposed in the previously mentioned approaches require larger amounts of data, reduction in data size, and removal of imbalance in the dataset can significantly affect replicability of results. ADReSS Challenge dataset includes data from 82 AD and 82 non-AD participants, of which 54 AD and 54 non-AD participants are included in the train set. The full-wave audio from each participant is further divided into an average of 24.86 (standard deviation *sd* = 12.84) normalized speech segments per participant using voice activity detection.

### 2.2. Classification Models and Approach

In this section, we'll briefly explain the language-based (transcript text input), acoustic feature-based and bi-modal models that we propose and progressively build on.

#### 2.2.1. Language-Based Models

**Figure 1:**
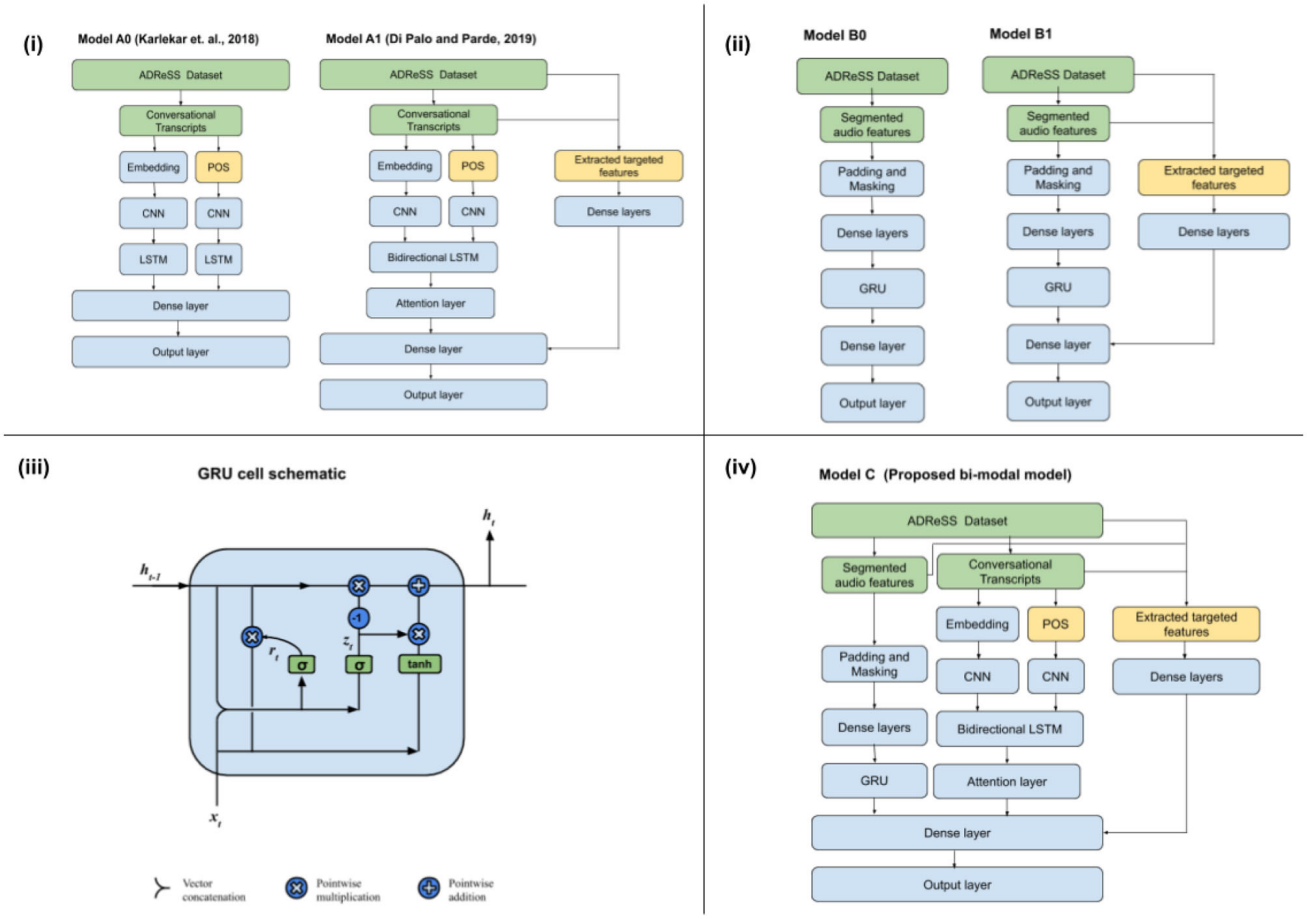
**(i)** Language-based models, **(ii)** speech-based models, **(iii)** GRU cell schematic, and **(iv)** bi-modal model for AD detection.

We first implement a CNN-LSTM model (Model A0) as proposed in Karlekar et al. (2018), which takes word embeddings (GloVe) as well as POS-tags as input, through two input streams, finally concatenated in a dense layer before passing it to the output layer. A dropout rate of 0.5 was used between the CNN and LSTM layer to prevent overfitting. We then implement the Model A1, as proposed by Di Palo and Parde (2019). It improves upon Model A0 by replacing the unidirectional LSTM in Model A0 with bidirectional LSTM layers with the insertion of attention mechanism on the hidden states of the LSTM and by including a dense neural network at the end of the LSTM layer to include targeted psycholinguistic, sentiment, and demographic features as described in Di Palo and Parde (2019). These targeted features are further explained in section 2.3. For models A0 and A1, we don't need to implement class weighting as done in Di Palo and Parde (2019) as ADReSS dataset doesn't have a class imbalance. Schematic representation of Models A0 and A1 can be found in Figure 1i.

#### 2.2.2. Acoustic-Feature Based Models

Similar to how previous models have proposed a recurrent unit based language processing stream which is later further enriched with targeted features, we propose a similar approach of using speech input stream and taking acoustic features into account, which is later enriched with relevant, targeted features. These acoustic features are extracted from audio segments. The Model B0 is comprised of a Speech-GRU, which is defined by a recurrent layer (GRU) which takes in audio segment features per from each speech segment while maintaining the temporal structure across segments as in the full-wave audio session. The goal of this GRU unit is to combine the features from the speech segments into a common vector while maintaining the temporal structure across segments. A schematic of the GRU cell is included in Figure 1iii. We also briefly experimented with the Model B0, by replacing the unidirectional GRU with bidirectional GRU layers with the insertion of attention mechanism on the hidden states of the GRU. But, since they do not improve the performance significantly, we continue with the Speech-GRU in our further study. In Model B1, we progressively build upon Model B0, by enriching the speech input processing stream with various AD specific features extracted from lengths of speech segments provided by voice activity detection (VAD) and disfluency and interventional features as well as idea density-based features from complete transcripts and full-wave audio. Schematic representation of Models B0 and B1 can be found in Figure 1ii.

#### 2.2.3. Bi-Modal Model

The Model that we propose is a direct combination of Model A1 and Model B1. The dense outputs from these two input streams is then concatenated and then connected to the output layer using dense connections. We use all targeted features from both the models in Model C. Schematic representation of Model C can be found in Figure 1iv.

### 2.3. Feature Extraction

In this subsection, we'll explain the targeted features used in Model A1, the acoustic feature sets used in Model B0, B1, C and the targeted features used in Model B1 and C. The targeted features used in Model A1, are token-level psycholinguistic features, token-level sentiment features and demographic features as described in Di Palo and Parde (2019). Each of the token-level features was averaged across all tokens in the instance, allowing us to obtain a participant-level feature vector to be coupled with the participant-level demographic features. The psycholinguistic features include (1) Age of acquisition of words which is the age at which a particular word is usually learned by individuals, (2) Concreteness which is a measure of word's tangibility, (3) Familiarity which is a measure of how often one might expect to encounter a word, (4) Imageability which is a measure of how easily a word can be visualized. Psycholinguistic features were obtained from an open-source repository^1^ based on the work of Fraser et al. (2016). Sentiment scores were based around measuring the word's sentiment polarity and were obtained using the NLTK's sentiment library. The demographic features include participants age at the time of the visit and gender.

We compare the use of different feature banks for acoustic feature extraction, namely *emobase, eGeMAPS* (Eyben et al., 2015), and *ComParE* (Eyben et al., 2013) on Model B0 and then use the best performing feature set in Model B1 and C. These acoustic feature sets are described as follows.

*emobase:* This feature set (Schuller et al., 2010) contains the mel-frequency cepstral coefficients (MFCC) voice quality, fundamental frequency (F0), F0 envelope, line spectral pairs (LSP), and intensity features with their first and second-order derivatives. Several statistical functions are applied to these features, resulting in a total of 1,582 features for every speech segment. Haider et al. (2019) and Luz et al. (2020) use an older emobase feature set of 988 features, whereas we use the newer emobase2010 set from the INTERSPEECH 2010 Paralinguistics Challenge (Schuller et al., 2010).

*eGeMAPS:* The eGeMAPS feature set (Eyben et al., 2015) is a result of attempts to reduce other feature sets to a basic set of 88 features with theoretical significance (Eyben et al., 2015). The eGeMAPS features thus have the potential to detect physiological changes in voice production. It contains the F0 semitone, loudness, spectral flux, MFCC, jitter, shimmer, F1, F2, F3, alpha ratio, Hammarberg index, and slope V0 features, as well as their most common statistical functional.

*ComParE:* The ComParE feature set (Eyben et al., 2013) includes energy, spectral, MFCC, and voicing related low-level descriptors (LLDs). LLDs include logarithmic harmonic-to-noise ratio, voice quality features, Viterbi smoothing for F0, spectral harmonicity, and psycho-acoustic spectral sharpness. Statistical functionals are also computed, bringing the total to 6,373 features.

We used OpenSMILE^2^ library for feature extraction using the emobase, eGeMAPS, ComParE feature bank. We performed a Pearson correlation test on the whole dataset to remove acoustic features that were significantly correlated with the segment duration (when *R* > 0.2). Hence, 72 eGeMAPS, 1,072 emobase, and 3,056 ComParE features were not correlated with the duration of the speech chunks and were therefore selected for the machine learning experiments. The purpose of this step is to remove acoustic features correlated with the segment duration to remove the “local” features which are independent of segment duration while training the Model B0 purely on the low-level acoustic features. We later add global features such as mean, median, and standard deviation of all the segment lengths in an interview while training Model B1. Local features which are highly correlated with the segment duration can at times act as unnecessary noise and lead the machine learning models to learn spurious correlations. This preprocessing step is common with the approach by Luz et al. (2020) and Haider et al. (2019).

Our Model B1 is an extension of our Model B0, enriched with targetted features and our Model C is a combination of Model A1 and B1 and thus Model C uses targetted features from both models A1 and B1. The additional targetted features used in Model B1 and then subsequently in Model C are specific to AD speech and are obtained from a combination of speech segments, full wave audio as well as manually generated transcripts. These targetted features specific to AD speech can be broadly split into three categories—speech segment length-based features, disfluency, and interventional rate-based features and the features based on the concept of idea density. It is important to note that these features are not captured by our Model B0. Segment length features include six statistics about speech chunks segmented by the VAD. Disfluency and interventional features include a set of six distinct features from the transcripts, such word rate, intervention rate, and different kinds of pause rates reflecting upon speech impediments like slurring and stuttering, which show up in the transcripts in forms of “umm,” “uhh” etc. Lastly, idea density based features comprise of the DEPID and DEPID-R features (Sirts et al., 2017) were computed as a measure of idea density. Idea density measures the rate at which ideas or elementary predications are expressed in an utterance or a text. Proportional idea density (PID) counts the expressed ideas and can be applied to any text. DEPID is a dependency-based method for computing PID and its version DEPID-R that enables to exclude repeating ideas which is a feature characteristic of AD speech.

### 2.4. Training and Validation Details

The following info is common to the training of all the models. We implement the models using Tensorflow 2.0 (Abadi et al., 2015). AdaGrad optimizer (Duchi et al., 2011) is used with a learning rate of 0.001. We train all the models for 200 epochs with early stopping as implemented in Di Palo and Parde (2019). All classification metrics use a classification threshold of 0.5.

The total dataset is split into a train dataset of 108 participants (54 AD and 54 non-AD participants) and test dataset of 48 participants (24 AD and 24 non-AD participants) as provided by Luz et al. (2020). Thus the test set is 30% of the total ADReSS dataset. K-fold cross validation (CV) is a useful CV strategy when sample size is lower as it uses every sample in the dataset but does not necessarily maintain balance in the labels (AD and non-AD) in each fold while splitting the train dataset into “k” folds. Performing a stratified k-fold CV assures this balance in labels in each fold and thus increases the reliability of metrics calculated on k-fold CV. We use 5-fold stratified cross-validation for all our models with the same seed value. We chose this cross-validation scheme over hold-out cross-validation schemes due to the small size of the dataset and to use every sample in the dataset. In Luz et al. (2020), the authors use leave one subject out (LOSO) cross-validation scheme, we find it infeasible in our case as training deep learning models are computationally more demanding and LOSO cross-validation scheme won't scale with more data without necessary compute requirements. For inference on test data, the models were trained on the complete train set for both the tasks separately and then tested on the test set.

## 3. Results

The outputs of a binary classification algorithm fall into one of the four categories—true positives *tp*, false positives *fp*, false negatives *fn* and true negatives *tn*, depending on whether the predicted label matches with the true label or not. Recall is also known as Sensitivity or the true positive rate. Then classification metrics are defined as follows,

(1) $$\begin{array}{l} \text{Precision}=\frac{tp}{tp+fp} \end{array}$$

(2) $$\begin{array}{l} \text{Recall}=\frac{tp}{tp+fn} \end{array}$$

(3) $$\begin{array}{l} \text{F}1\text{ score}=2\frac{\left(\text{precision}\right)\left(\text{recall}\right)}{\text{precision}+\text{recall}} \end{array}$$

In context of reproducing results by Karlekar et al. (2018) and Di Palo and Parde (2019) on ADReSS dataset, the classification task results (Precision, Recall, F1 score, and Accuracy) are shown in Table 1 for 5-fold cross-validation and test setting, respectively. The results show that Model A1 performs better than Model A0 in all aspects of the classification task. We notice the difference between AD classification accuracy (0.8384 and 0.8820, respectively) achieved in Karlekar et al. (2018) and Di Palo and Parde (2019) on the complete Dementia Bank dataset and the AD classification accuracy achieved (0.6875 and 0.7292, respectively) by re-implementing those methods on ADReSS dataset.

**Table 1:** Validation and Test results of the language based models on the classification task.

**Model**	**Val/Test**	**Class**	**Recall**	**Precision**	**F1 score**	**Accuracy**
A0 (Karlekar et al., 2018)	5-fold CV	Non-AD	0.811 ± 0.085	0.637 ± 0.071	0.710 ± 0.059	0.673 ± 0.065
		AD	0.539 ± 0.121	0.752 ± 0.081	0.619 ± 0.090	
	Test set	Non-AD	0.8333	0.6451	0.7272	0.6875
		AD	0.5416	0.7647	0.6341	
A1 (Di Palo and Parde, 2019)	5-fold CV	Non-AD	0.836 ± 0.202	0.706 ± 0.152	0.735 ± 0.072	**0.710** ± **0.067**
		AD	0.600 ± 0.241	0.866 ± 0.167	0.654 ± 0.113	
	Test set	Non-AD	0.9167	0.6667	0.7719	**0.7292**
		AD	0.5416	0.8667	0.6667	

*Bold values represent the validation and test accuracies of best performing model amongst the models under consideration in the respective table*.

In the context of the proposed acoustic feature processing Speech-GRU, the classification task results with the use of different acoustic feature set are shown in Table 2 for 5-fold cross-validation and test sets, respectively. We observe that our model B0 with use of emobase as the acoustic feature set performs best followed by eGeMAPS and we observe that our recurrent model with ComParE features as input fails to learn. Our model B0 with the feature set emobase performs better than the acoustic feature-based baseline accuracy of 0.62 set by Luz et al. (2020). We use the best performing feature set (emobase) further, for our models B1 and C. We further also experimented with Speech-GRU in model B0 (emobase feature set) by replacing GRU layer with a bidirectional GRU layer followed by the use of attention mechanism, but it resulted in validation accuracy of 0.6632 ± 0.0368 which did not significantly better than our basic Speech-GRU stream. Since we did not observe a significant improvement, we use our plain GRU stream for acoustic feature processing in models B1 and C.

**Table 2:** Validation and Test results of the Model B0 with different feature sets on the classification task.

**Feature set**	**Val/Test**	**Class**	**Recall**	**Precision**	**F1 score**	**Accuracy**
eGeMAPS	5-fold CV	Non-AD	0.527 ± 0.120	0.710 ± 0.151	0.581 ± 0.058	0.635 ± 0.034
		AD	0.745 ± 0.156	0.618 ± 0.029	0.667 ± 0.058	
	Test set	Non-AD	0.7500	0.5625	0.6428	0.5833
		AD	0.4166	0.6250	0.5	
emobase	5-fold CV	Non-AD	0.659 ± 0.094	0.704 ± 0.168	0.663 ± 0.057	**0.665** ± **0.082**
		AD	0.673 ± 0.219	0.664 ± 0.049	0.652 ± 0.125	
	Test set	Non-AD	0.6667	0.6400	0.6530	**0.6458**
		AD	0.6250	0.6521	0.6382	
ComParE	5-fold CV	Non-AD	0.441 ± 0.176	0.534 ± 0.139	0.475 ± 0.148	0.533 ± 0.129
		AD	0.625 ± 0.144	0.538 ± 0.132	0.573 ± 0.124	
	Test set	Non-AD	0.5833	0.5185	0.5490	0.5208
		AD	0.4583	0.5238	0.4888	

*Bold values represent the validation and test accuracies of best performing model amongst the models under consideration in the respective table*.

The classification task results for the models B1 and C are shown in Table 3. Our results show that model B1, enriched with targeted features performs better than model B0 with an accuracy of 0.6875 on the test set. We further conduct ablation experiments on model B1 to tease out which of these targeted features contribute the most. The results of our ablation experiment in Table 4 show that none of the targeted features (segment length based, disfluency, and interventional rate based and idea-density based) individually improve the test results of model B1, in comparison to model B0. But all of these features combined improve the classification accuracy of our model B1. Our model C benefits from linguistic feature processing stream of model A1 but does not perform better than model A1 in terms of test or validation accuracy. We notice a significant improvement in AD class Recall and a reduction in AD class Precision from model A1 to model C.

**Table 3:** Validation and Test results of the Model B1 and Model C on the classification task.

**Model**	**Val/Test**	**Class**	**Recall**	**Precision**	**F1 score**	**Accuracy**
B1	5-fold CV	Non-AD	0.662 ± 0.175	0.670 ± 0.101	0.652 ± 0.125	0.662 ± 0.109
		AD	0.666 ± 0.170	0.675 ± 0.126	0.659 ± 0.139	
	Test set	Non-AD	0.8333	0.6452	0.7272	0.6875
		AD	0.5416	0.7647	0.6341	
C	5-fold CV	Non-AD	0.778 ± 0.104	0.673 ± 0.092	0.715 ± 0.070	**0.693** ± **0.082**
		AD	0.615 ± 0.151	0.743 ± 0.097	0.659 ± 0.112	
	Test set	Non-AD	0.8333	0.6896	0.7547	**0.7292**
		AD	0.6250	0.7894	0.6976	

*Bold values represent the validation and test accuracies of best performing model amongst the models under consideration in the respective table*.

**Table 4:** Ablation experiments with Model B1 with different targeted features; Test results on classification task.

**Targeted features**	**Class**	**Recall**	**Precision**	**F1 score**	**Accuracy**
Seglen	Non-AD	0.5833	0.6363	0.6087	0.6250
	AD	0.6667	0.6154	0.6400	
Disf-inv	Non-AD	0.5000	0.6000	0.5454	0.5833
	AD	0.6667	0.5714	0.6154	
DEPID	non-AD	0.6250	0.6522	0.6383	0.6458
	AD	0.6667	0.6400	0.6530	
All combined	non-AD	0.8333	0.6452	0.7273	**0.6875**
	AD	0.5416	0.7647	0.6341	

*Bold values represent the validation and test accuracies of best performing model amongst the models under consideration in the respective table*.

**Figure 2:**
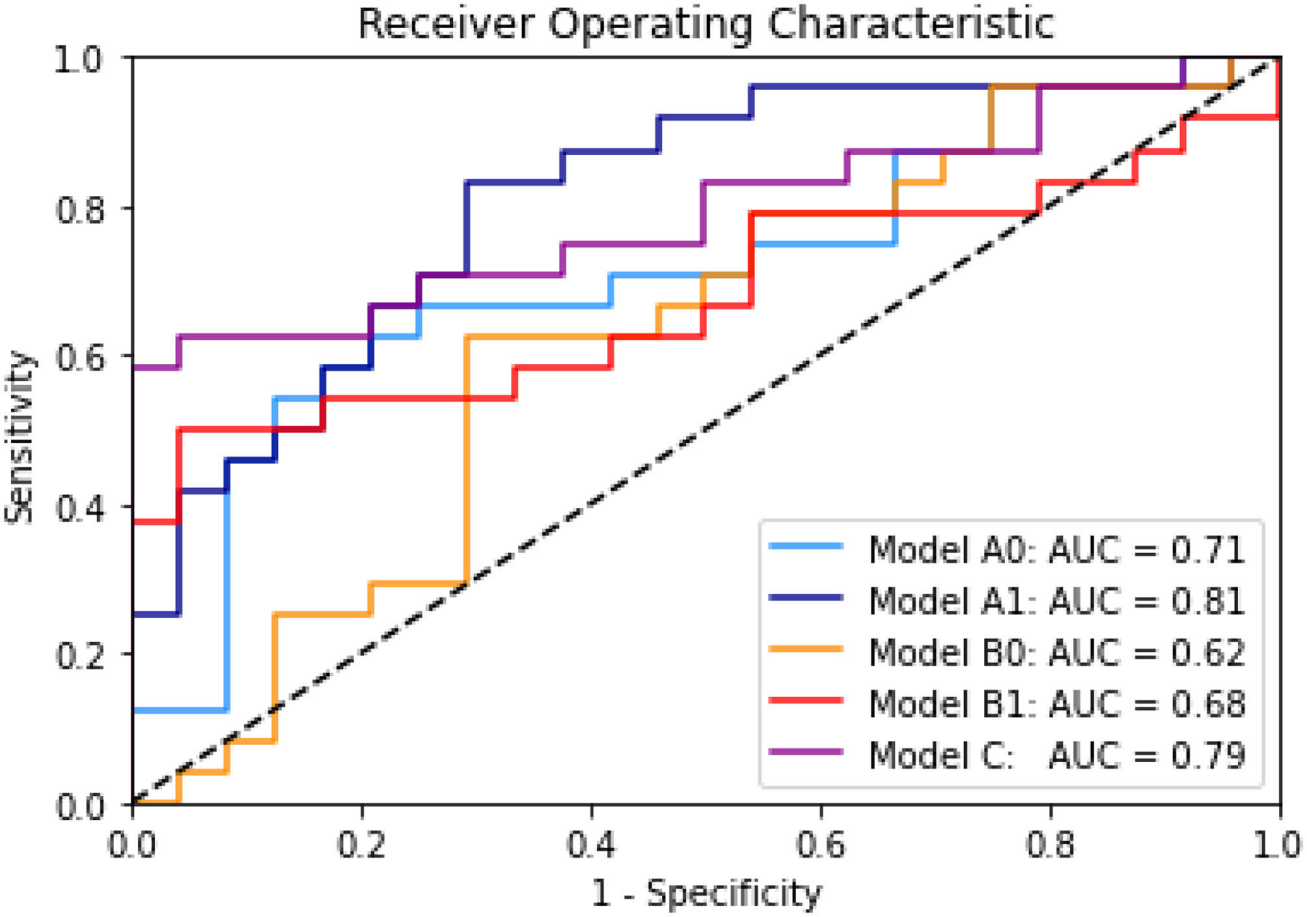
Receiver operating characteristics for all Models A0, A1, B0, B1, and C and the area under the curve (AUC). Results on test set.

Finally, we include the Area under the Receiver-Operator characteristic curve for all the models in the Figure 2 for quick comparison of the performance of all the models on the test set.

## 4. Discussion

**Figure 3:**
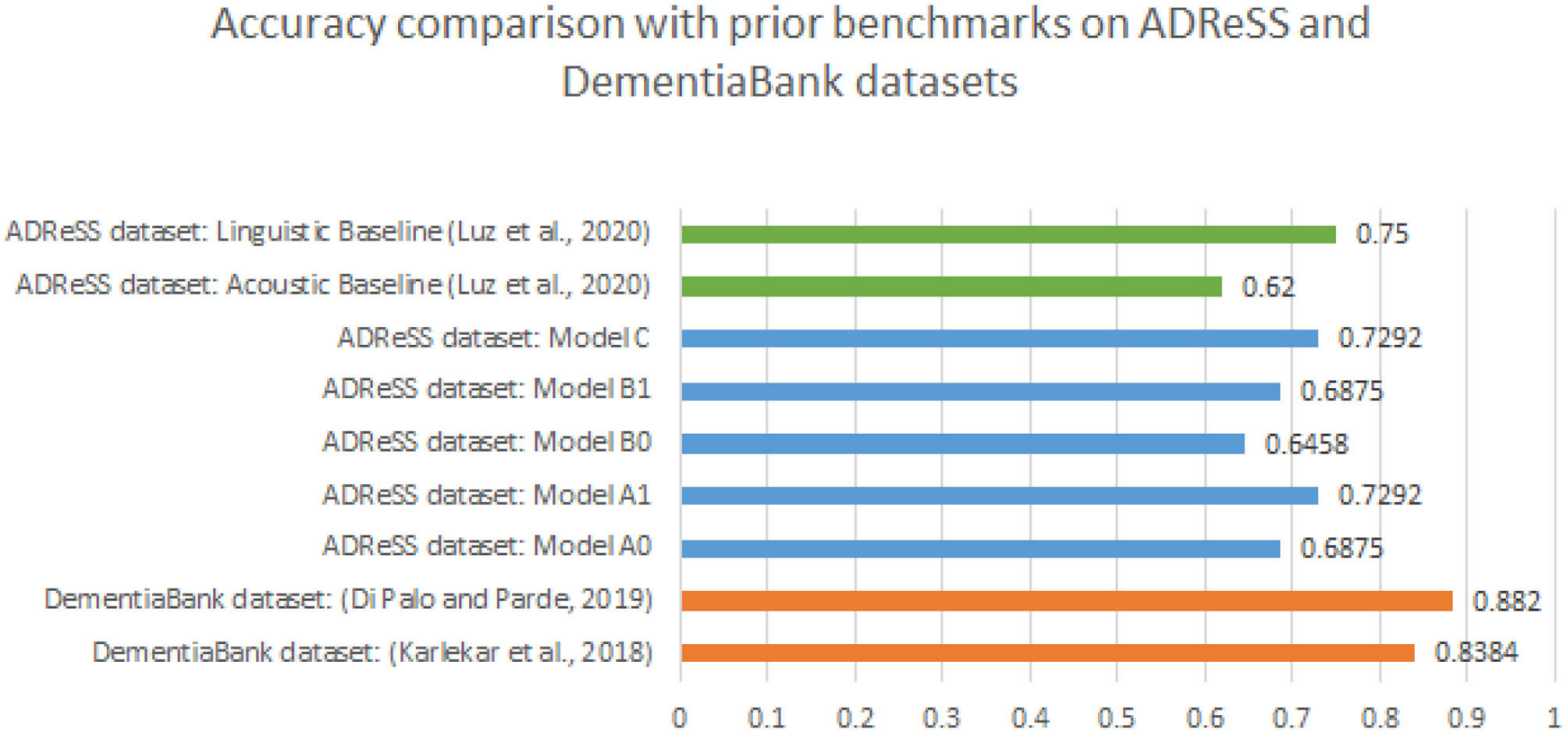
Accuracy comparison of all Models A0, A1, B0, B1, and C with baselines on ADReSS dataset as well as referred state of the art approaches on DementiaBank dataset.

Amongst language-based models, the improvement in performance from model A0 to A1 can be attributed to the use of attention as well as the use of psycholinguistic and sentiment features. As per our results, model A0 and A1 which have shown the state of the art results on the complete Dementia bank dataset don't perform better than the linguistic feature baseline set by Luz et al. (2020) of accuracy 0.75 on the ADReSS dataset. This is important to note because the primary motivation of Karlekar et al. (2018) was to develop end to end deep learning method for AD detection with minimal feature engineering. Furthermore, noticing the difference in accuracy and F1 scores, there could be multiple factors involved in the success of Karlekar et al. (2018) and Di Palo and Parde (2019) and those that hinder the replicability of results on ADReSS dataset. The most prominent factor being, repeated occurrences of samples from the same participant in the Dementia Bank dataset. This could lead to significant overfitting to participant dependent features in models trained the DementiaBank dataset. As explained in section 2.1, DementiaBank has 243 transcripts from 104 non-AD participants whereas 1,049 transcripts from 208 AD participants. In comparison to that, ADReSS dataset includes only one transcript and full wave audio per participant, with 54 AD and 54 non-AD participants in the train set and 24 AD and 24 non-AD participants in the test set. Thus the total number of samples in DementiaBank is 1,292, which is around 8 times the dataset size of ADReSS. ADReSS dataset allows us to test the speaker independent nature of previously proposed models and our new model as there are no multiple sessions per participant. It is evident from other success of deep learning methods in other domains (not specific to AD speech) that such methods do scale with data, but that need not necessarily apply to tasks such as early detection of AD. Thus, we cannot take a purely minimal feature engineering approach, and future work should instead focus more on developing and utilizing features relevant to AD speech. Benchmarking on a dataset with more subjects in the future would help build a better understanding of whether these methods perform better compared to complete manual feature engineering-based solutions or not. Accuracy comparison of all the models with baselines on ADReSS dataset by Luz et al. (2020) as well as results on the DementiaBank dataset by Karlekar et al. (2018) and Di Palo and Parde (2019) can be found in Figure 3.

Our results from Table 2 help us answer the question whether there exist temporal patterns relevant to AD detection in the acoustic features extracted using these feature sets emobase, eGeMAPs, ComParE etc. which are not explicitly designed for AD speech. Amongst the three feature sets, we observe that our Speech-GRU does pickup some relevant temporal patterns and effectively combines these features into a common vector. Our Speech-GRU with emobase feature set also performs better than the baseline by Luz et al. (2020), which takes the maximum vote of classification output of each of the speech segment. Still, the improvement is relatively small (2%). Moreover, the use of attention did improve the performance in language-based model A1, suggesting that there are temporal patterns which are relevant to AD speech in word vectors and POS-tags. But the same approach did not improve the performance in Speech-GRU, suggesting a general lack of temporal patterns across paralinguistic features of the speech segments. Future work could benefit from the development of AD specific feature sets.

It is important to note that our performance of model B0 is representative of the performance of AD detection without the use of any manual transcription. All the transcripts in Dementia Bank and ADReSS dataset are manually generated, and deploying this service would instead require automated transcription. Readers can refer to Zayats et al. (2019) for detailed analysis of impact of transcription errors (manual and automated) on automatic disfluency detection. Various disfluency and interventional features in our approach, as well as other state of the art approaches, rely on these manually generated transcripts for feature extraction and their performance may vary depending on whether the transcription is automated or not. In the ablation experiments, the decrease in the test accuracy in case of enrichment with disfluency and interventional features could be as these word rates, interventional rates, pause rates were extracted from manual transcripts. A better approach could be using forced alignment tools to get precise disfluency features, but since not all samples in the ADReSS dataset aligned with the transcript text, we didn't explore that idea further.

We observe that the language-based models A0 and A1 are characterized by higher non-AD class recall scores and higher AD class precision scores which are further aggravated from model A0 to A1. We observe that speech-based models were generally characterized by nearly equal precision and recall scores in AD and non-AD classes and we can also observe similar influence in the model C.

There are two possible reasons for the bimodal model C not performing significantly better than the language-based model A1, which are explained as follows. The first is that, the inherent representations learnt by the recurrent stream in Model A1 (trained on word embeddings and POS tags) and in Model B1 (on acoustic features of each segment, in lieu of acoustic embeddings) are quite different. And a mere concatenation of the final layers, can be thought of as a linear combination of the two representations and we observe that it does not provide rich space for a variety of cross-dimensional and non-linear combinations among the two representations. Because of this, the outputs of a Model B1 (which is a relatively weak learner in comparison to it's language counterpart Model A1) can act as noise in linear combination of these representations. This problem has been addressed by a variety of trainable feature aggregation methods, especially visual and language based representations, in the context of multimodal emotion detection or sentiment analysis. One of the most promising solution, which has proven to be successful in the context of multimodal sentiment analysis is focusing on word-level fusion (Chen et al., 2017), where they align the words with the speech segment of each word and generate combined Gated Multimodal Embeddings (GME), rather than combine the two representations in the final layers as we do in Model C. We believe a similar approach to generating combined word-level embeddings, where influence of each modality is also learnable through gating, can also help in the context of AD speech. Unfortunately, word-level fusion methods require alignment of both the modalities, which is very expensive in terms of reduction in data size as not all samples align even with the state of the art methods. Though this is a feasible option for other problems such as sentiment analysis, where data is in abundance and where the study can be carried out with a fraction of aligned data. But it's not a feasible option in small sized datasets like the ADReSS dataset as we observed while running the alignment tools (Montreal Forced Aligner^3^), <70% of the full wave audio samples aligned with the manually generated transcripts. The speech segment chunks provided by the ADReSS dataset use voice activity detection (VAD) and often include multiple words rather than providing a word-to-word alignment thus cannot be used for creating multimodal word embeddings. Readers can refer to Baltrušaitis et al. (2018) or a detailed survey of approaches and challenges faced in multi-modal machine learning in terms of representation, alignment, and fusion. Future work, in availability of more data, can attempt similar approaches to AD detection.

**Figure 4:**
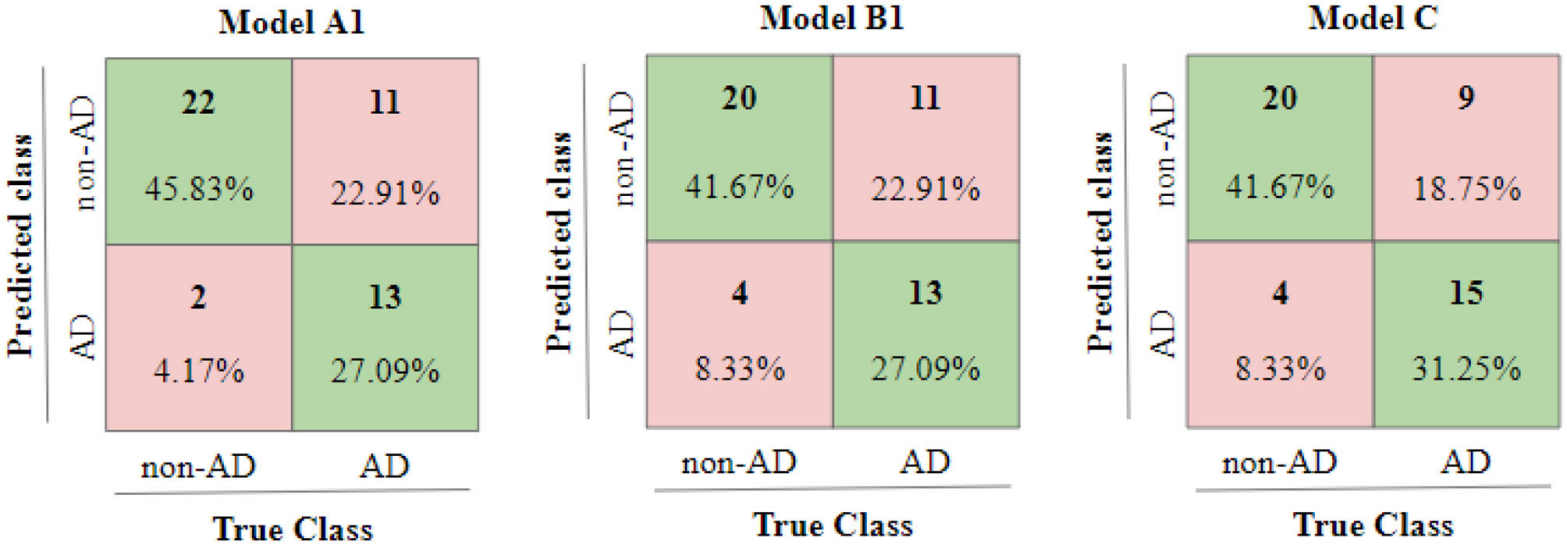
Confusion matrices of Test results of Model A1, B1, and C.

The second reason is that the idea density features used in Model B1 and then subsequently in Model C, have been computed using the transcripts. The disfluency and interventional rates used are also obtained from transcripts in lieu of aligning speech with transcripts. We compute the similarity in predictions of two models as ratio of predictions which match between two models upon total predictions in the test set (i.e., 48). We find the similarity between predictions of Model A1 and Model C to be 0.6667 whereas the similarity in predictions of model B1 and model C to be 0.5416. Furthermore, we also observe that the similarity between predictions of Model A1 and B1 is 0.6667 and is greater than similarity between predictions of Model A1 and Model B0 which is 0.5834, suggesting that the additional targeted features obtained from transcripts and used in Model B1 might have already been captured in the Model A1 which trained only on the transcript data. Apart from the similarities in predictions, we can observe the confusion matrices of test predictions of Model A1, B1, and C in Figure 4 which show the influence of Model A1 and B1 on Model C.

## 5. Conclusions

We re-implement existing deep learning-based methods on ADReSS dataset and discuss the challenges of the approach. We also introduce a bi-modal deep learning approach to AD classification from spontaneous speech and study in detail the Speech-GRU stream, which is further enriched with AD specific features through comprehensive comparisons of different variants. An important finding of this study is that the addition of targeted features increases the performance in AD detection in both language-based and acoustic-based models. Though the speech-GRU stream in our bi-modal approach is a relatively weaker learner compared to the language-based counterparts in the network, future work can aim at improving the acoustic feature extraction as well as a better combination of representations from different modalities. The Speech-GRU without and with extra targeted features performs much better than acoustic baselines and Model B0 is also representative of the extent of performance of solutions which don't rely on manual transcription. Our results help us answer questions regarding the existence of temporal patterns relevant to AD detection in para-linguistic acoustic features often extracted using common feature sets as well as also address the reasons for a drop in accuracy of models on ADReSS dataset which were previously state of the art approaches on the complete Dementia Bank dataset.

## Data Availability Statement

Publicly available datasets were analyzed in this study. This data can be found at: http://www.homepages.ed.ac.uk/sluzfil/ADReSS/.

## Author Contributions

PM participated in the ADReSS challenge, developed, and trained the machine learning models. VB helped in manuscript preparation and supervised the study. All authors contributed to the article and approved the submitted version.

## Conflict of Interest

The authors declare that the research was conducted in the absence of any commercial or financial relationships that could be construed as a potential conflict of interest.

## Footnotes

^1^https://github.com/vmasrani/dementia_classifier.

^2^https://www.audeering.com/opensmile/.

^3^https://github.com/MontrealCorpusTools/Montreal-Forced-Aligner.
